# Increased serum fetuin-B concentration is associated with HOMA-β and indices of liver steatosis in women with polycystic ovary syndrome: a pilot study

**DOI:** 10.1530/EC-19-0243

**Published:** 2019-07-15

**Authors:** Agnieszka Adamska, Aleksandra Maria Polak, Anna Krentowska, Agnieszka Łebkowska, Justyna Hryniewicka, Monika Leśniewska, Irina Kowalska

**Affiliations:** 1 Department of Endocrinology, Diabetology and Internal Medicine, Medical University of Białystok, Białystok, Poland; 2 Department of Internal Medicine and Metabolic Diseases, Medical University of Białystok, Białystok, Poland; 3 Department of Reproduction and Gynecological Endocrinology, Medical University of Białystok, Białystok, Poland

**Keywords:** fetuin-B, PCOS, HOMA-β, liver steatosis

## Abstract

**Objective:**

PCOS women are characterized by insulin resistance and have higher tendency to the development of hepatic steatosis. Fetuin-B has been introduced as a hepatokine/adipokine, which is increased in hepatic steatosis and may be connected with glucose metabolism disturbances. The aim of the study was to evaluate the relationships between serum fetuin-B concentration and indices of insulin resistance, insulin secretion and markers of liver steatosis in PCOS women in comparison to the control group.

**Patients and methods:**

The study group included 108 women – 57 women with PCOS and 51 women matched for age and BMI as a control group. Serum concentration of fetuin-B was estimated. Homeostasis model assessment of insulin resistance (HOMA-IR) and homeostasis model assessment β cell function (HOMA-β) were calculated. Fatty liver index (FLI), lipid accumulation product (LAP) and visceral adiposity index (VAI) were used as markers of liver steatosis.

**Results:**

We found higher serum concentration of fetuin-B and FLI in PCOS women in comparison to the control group (all *P* < 0.05). We observed a positive relationship between serum fetuin-B concentration and HOMA-β (*r* = 0.43, *P* = 0.01), HOMA-IR (*r* = 0.31, *P* = 0.01), FLI (*r* = 0.29, *P* = 0.02), VAI (*r* = 0.29, *P* = 0.02) and LAP (*r* = 0.32, *P* = 0.01) in PCOS women. We also noticed a relationship between HOMA-IR and FLI (*r* = 0.42, *P* = 0.01), VAI (*r* = 0.38, *P* = 0.004) and LAP (*r* = 0.41, *P* = 0.001) in this group. Multiple regression analysis revealed that HOMA-β (β = 0.39, *P* = 0.002) and LAP (β = 0.27, *P* = 0.02) were independently connected with serum fetuin-B levels in women with PCOS.

**Conclusions:**

Serum fetuin-B levels are higher in women with PCOS and are independently connected with HOMA-β and hepatic steatosis.

## Introduction

Polycystic ovary syndrome (PCOS) is known as the most common endocrinological disorder and affects up to 20% of premenopausal women (1). PCOS is characterized by clinical and/or biochemical hyperandrogenism, ovulatory dysfunction and characteristic image of the ovaries in the ultrasound (2). A lot of data indicated that insulin resistance is a key factor in the development of metabolic disturbances in PCOS, for example, obesity, type 2 diabetes (T2D), cardiovascular disease (3) and non-alcoholic fatty liver disease (NAFLD) (1).

NAFLD is histologically defined by the presence of steatosis in at least 5% of liver tissue and is the most common reason of chronic liver disease in many parts of the world (4). The occurrence of NAFLD in women with PCOS is more prevalent than in control group (50.6 vs 34.0%) (5). Furthermore, data have shown that these women are more likely to have more severe forms of NAFLD (4). Accumulation of fat in the liver is responsible for NAFLD, as well as for insulin resistance. However, pathogenesis of NAFLD is multifactorial (6).

Fetuin-B is a member of the fetuin family, part of the cystatin superfamily of cysteine protease inhibitors (7) and is predominantly expressed in the liver (8, 9), but also, to a lesser extent, in other tissues, such as placenta (10), white adipose tissue and heart (8). It has been shown that fetuin-B could be activated by agonists of farnesoid X receptor, which is a key factor in the regulation of lipid and carbohydrate metabolism (11). In an experimental study, Meex *et al*. showed that fetuin-B impaired insulin action in myotubes and hepatocytes and caused glucose intolerance in mice, whereas knockdown of this protein in obese mice was connected with improved glucose tolerance (8). In human studies, it has been demonstrated that serum fetuin-B was increased in humans with liver steatosis (12), patients with T2D, women with gestational diabetes mellitus (GDM) (13), as well as in patients with coronary artery disease, in comparison to healthy controls (14).

Fetuin-B appears to be a hepatokine that becomes dysregulated in hepatic steatosis and insulin resistance. However, no evidence regarding the association between fetuin-B, insulin resistance and hepatic steatosis in PCOS women is currently available.

Therefore, the aim of the present study was to evaluate the relationships of serum fetuin-B concentration with indices of insulin resistance and insulin secretion, and with markers of liver steatosis in PCOS women in comparison to the control group.

## Subjects and methods

### Subjects

A prospective, cross-sectional study was conducted between January 2017 and May 2018. The study group included 108 women – 57 women with PCOS and 51 women matched for age and BMI (23.9 (18.8–35.0) kg/m^2^ vs 22.8 (19.2–32.9) kg/m^2^) as a control group. Women with PCOS were recruited from the Department of Endocrinology, Diabetology and Internal Medicine, Medical University of Białystok and among students. The control group were recruited from the staff and students. All women were non-smoking and did not drink more than 20 g of alcohol per day. PCOS was diagnosed according to the 2003 Rotterdam ESHRE/ASRM PCOS Consensus Workshop Group diagnostic criteria (15). PCOS was recognized when at least two of the following three criteria were present: (1) clinical and/or biochemical hyperandrogenism, (2) oligomenorrhoea or anovulation, (3) polycystic ovaries on ultrasound (>12 follicles measuring 2–9 mm in diameter or ovarian volume >10 mL in at least one ovary). The exclusion criteria for PCOS group and control group were history of known liver disease (i.e., hepatitis B and C, autoimmune, genetic and drug-induced diseases), thyroid disorders (hypothyroidism, hyperthyroidism), morbid obesity, cardiovascular disease, hyperlipidemia; other causes of irregular menstrual cycles and/or androgen excess (i.e., hyperprolactinemia, Cushing’s syndrome, late-onset congenital adrenal hyperplasia, or diseases of the adrenal glands, pregnancy and breastfeeding); type 1 or type 2 diabetes; chronic or acute infection (within the previous 30 days); any other serious medical problem; hormonal contraception and/or anti-androgen therapy (within the previous 6 months). Moreover, participants taking any medications (e.g., drugs affecting lipid and glucose metabolism) were also excluded from the study. All the patients participating in the study were Caucasians. All persons gave their informed consent prior to their inclusion in the study after full explanation of the purpose and nature of all procedures used. The study protocol was approved by the Ethics Committee of the Medical University of Białystok and was concordant with the Declaration of Helsinki.

### Study protocol

All study participants – PCOS women and controls – followed the same study protocol. Clinical examination was performed in all women. Clinical hyperandrogenism was evaluated using the modified Ferriman–Gallwey score for hirsutism (more than eight points was considered as clinical hyperandrogenism) and/or presence of acne. Oligo/amenorrhea and anovulation were considered when women had fewer than six menses during the previous year. Ultrasound scans were done for all the patients by the same gynecologist with a 5–9 MHz transvaginal transducer (Voluson 730 Expert, GE Healthcare) in the early follicular phase of the menstrual cycle. Ovarian volume was calculated using the simplified formula for a prolate ellipsoid (16). All analyses were carried out after an overnight fast. Studies were performed in the PCOS group 3–5 days after a spontaneous menses or independent of cycle phase in the presence of amenorrhea. In the control group, the studies were performed during the early follicular phase (3–5 days) of their menstrual cycles. All subjects underwent an oral glucose tolerance test (OGTT) with 75 g of glucose.

### Anthropometric measurements

BMI was calculated as body weight in kilograms divided by height in meters squared (kg/m^2^). Waist circumference was measured at the smallest circumference between the rib cage and the iliac crest, with the subject in the standing position. The hip circumference measurement was obtained at the maximum perimeter at the level of the femoral trochanters. Systolic and diastolic blood pressure was recorded. Fat mass (%) and fat free mass (kg) was estimated by multi‐frequency bioelectrical impedance analysis using the InBody 770 Body Composition Analyzer (Biospace, Beverly Hills, CA, USA).

### Biochemical analyses

Plasma glucose level was measured immediately by the enzymatic reference method with hexokinase (Cobas c111, Roche Diagnostic). Serum insulin concentration was assayed by immunoradiometric method (DIAsource ImmunoAssays, Louvain-la-Neuve, Belgium). The minimum detectable concentration was 1 µIU/mL and the intra-assay and inter-assay coefficients of variation (CVs) were below 2.2 and 6.5%, respectively. In this method, human and animal proinsulins present no cross-reactions. Plasma total cholesterol (TC), high-density lipoprotein cholesterol (HDL-C) and triglycerides (TG) were assessed by the enzymatic methods using commercial kits produced by ANALCO-GBG (Warsaw, Poland). Plasma low-density lipoprotein cholesterol (LDL-C) was calculated according to the Friedewald’s formula. Serum FSH, LH, prolactin and total testosterone concentrations were measured by the immunoradiometric method (DIAsource ImmunoAssays, Belgium). Serum sex hormone–binding globulin (SHBG) was measured by immunoradiometric assay (ZenTech, Angleur, Belgium). Free androgen index (FAI) was calculated as serum total testosterone (nmol/L) × 100/SHBG (nmol/L) ratio. Serum gamma-glutamyl transferase (GGT) was assessed with colorimetric method (Cobas c111, Roche Diagnostic).

Serum concentration of fetuin-B was estimated with ELISA Kit (Cloud Clone, Wuhan, China), following the manufacturer’s protocol. The degree of precision of the ELISA system in terms of coefficient of variance (%) of intra-assay and that of inter-assay was less than 10.0 and 12.0%, respectively. Moreover, the ELISA was specific for human fetuin-B and did not cross-react with human fetuin A or human cystatin C.

### Calculations

Homeostasis model assessment of insulin resistance (HOMA-IR) was calculated according to the formula: (fasting insulin (µIU/mL) × fasting plasma glucose (mmol/L))/22.5 (17).

Homeostasis model assessment β cell function was calculated according to the formula: 20 × fasting insulin (µIU/ml)/(fasting plasma glucose (mmol/L) − 3.5) (17).

Fatty liver index (FLI), lipid accumulation product (LAP) and visceral adiposity index (VAI) were used as markers of NAFLD. FLI was calculated according to the algorithm described by Bedogni *et al*. (18), based on BMI, waist circumference, plasma TG and serum GGT. LAP was calculated using waist circumference and plasma TG concentrations according to the formula for women by Bedogni *et al*. (19). VAI was calculated using BMI, waist circumference, plasma TG and HDL cholesterol as formulated by Amato *et al*. (20).

### Statistical analysis

Statistical analyses were performed using STATISTICA 10.0 software. Before the analyses were carried out, the distribution of the variables was tested for normality using Shapiro–Wilk W test and non-normally distributed parameters were logarithmically transformed. The differences between clinical and biochemical parameters between PCOS group and control women were evaluated with an unpaired Student’s *t* test. Continuous variables are presented as mean ± standard deviation (s.d.). The relationships between the variables were evaluated with Spearman’s correlation coefficient. Afterwards, multivariate regression analysis was performed to identify independent relationships. The level of significance was accepted at *P* < 0.05.

## Results

The clinical and biochemical characteristics of the studied groups are shown in Table 1. The PCOS and control group did not differ in age, anthropometric indices, HOMA-IR, HOMA-β, plasma lipids, as well as plasma glucose concentrations at 0 min and 120 min of OGTT (all *P* > 0.05). We found higher plasma glucose concentration at 60 min of OGTT and higher serum level of insulin at 120 min of OGTT in the PCOS group in comparison to the control group (*P* = 0.003, *P* = 0.03; respectively) (Table 1).

**Table 1:** Clinical and biochemical characteristics of the studied groups.

	Control group (*n* = 51)	PCOS (*n* = 57)	*P* value
Age (years)	26.3 ± 4.3	25.6 ± 4.7	0.40
BMI (kg/m^2^)	22.8 ± 3.1	23.9 ± 3.7	0.07
Waist circumference (cm)	79.5 ± 8.3	82.2 ± 10.0	0.13
Hip circumference (cm)	98.7 ± 7.1	99.4 ± 8.1	0.60
Fat free mass (kg)	44.8 ± 3.7	44.8 ± 5.0	0.78
Fat mass (%)	28.9 ± 6.3	31.7 ± 8.6	0.06
Follicle-stimulating hormone (IU/L)	5.4 ± 1.7	5.4 ± 1.4	0.90
Luteinizing hormone (IU/L)	4.1 ± 1.2	5.1 ± 2.3^a^	0.01
Total testosterone (ng/mL)	0.56 ± 0.1	0.75 ± 0.4^a^	0.01
SHBG (nmol/L)	66.0 ± 30.2	56.5 ± 46.1	0.21
FAI	3.4 ± 2.5	6.8 ± 6.2^a^	<0.01
Prolactin (ng/mL)	15.5 ± 14.0	16.2 ± 13.4	0.79
Glucose 0′ OGTT (mg/dL)	94.1 ± 9.5	92.0 ± 7.1	0.18
Glucose 60′ OGTT (mg/dL)	97.7 ± 20.5	115 ± 36.5^a^	0.003
Glucose 120′ OGTT (mg/dL)	88.8 ± 16.6	95.8 ± 22.2	0.06
Insulin 0′ OGTT (µIU/mL)	10.0 ± 4.8	9.7 ± 4.0	0.72
Insulin 60′ OGTT (µIU/mL)	55.9 ± 45.8	71.0 ± 50.5	0.10
Insulin 120′ OGTT (µIU/mL)	30. ± 18.3	46.9 ± 53.2^a^	0.03
HOMA-IR	2.2 ± 1.1	2.3 ± 1.0	0.65
HOMA-B	125 ± 70	125 ± 56	0.11
Total cholesterol (mg/dL)	175 ± 28.1	169 ± 25	0.23
HDL-cholesterol (mg/dL)	65.7 ± 20.7	71.4 ± 16.4	0.11
LDL-cholesterol (mg/dL)	91.5 ± 24.3	89.6 ± 21.6	0.67
TG (mg/dL)	62.0 ± 28.0	69.3 ± 39.6	0.27
Fetuin-B (µg/mL)	79.2 ± 16.7	88.1 ± 26.7^a^	0.04
VAI	0.78 ± 0.48	1.01 ± 1.04	0.15
LAP	15.7 ± 10.7	21.0 ± 22.7	0.13
FLI	8.0 ± 10.2	14.4 ± 19.6^a^	0.02
GGT	9.4 ± 2.7	13.1 ± 8.2^a^	0.02

Data are presented as mean ± s.d. The level of significance was accepted at ^a^*P* < 0.05.

BMI, body mass index; FAI, free androgen index; FLI, fatty liver index; GGT, gamma-glutamyl transferase; HOMA-B, homeostatic model assessment β cell function; HOMA-IR, homeostasis model assessment of insulin resistance; LAP, lipid accumulation product; OGTT, oral glucose tolerance test; PCOS, polycystic ovary syndrome; SHBG, serum sex hormone–binding globulin; TG, triglycerides; VAI, visceral adiposity index.

Women with PCOS had higher serum LH concentration (*P* = 0.01), as well as serum concentrations of total testosterone (*P* = 0.01) and FAI (*P* < 0.01) as compared to control group (Table 1).

**Figure 1:**
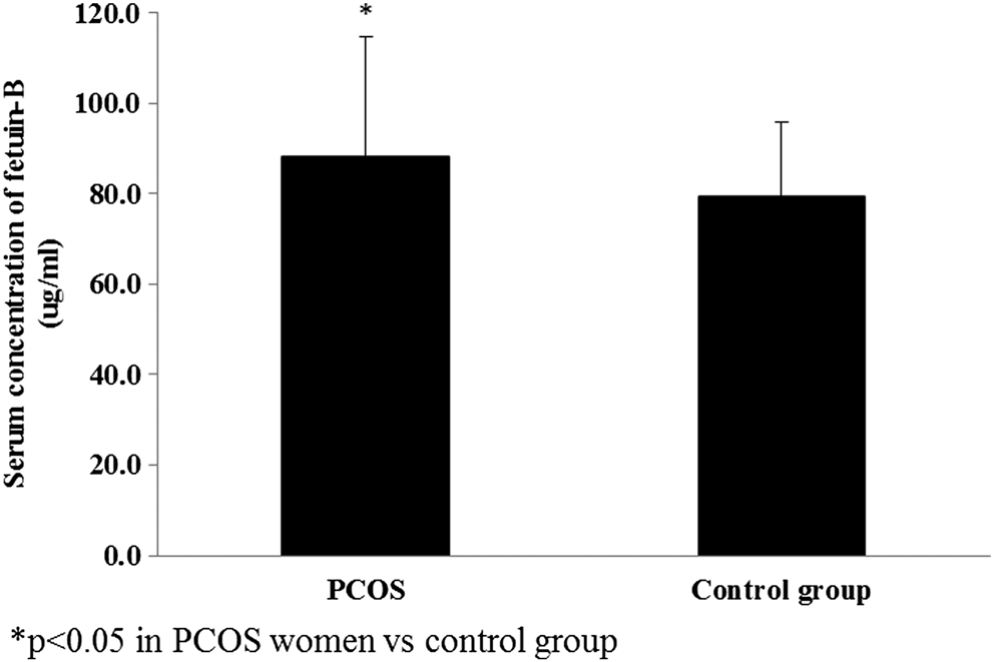
Serum concentration of fetuin-B in PCOS women and control group (*P* = 0.04).

We found that serum fetuin-B concentration (*P* = 0.04), GGT (*P* = 0.02) and FLI (*P* = 0.02) were higher in the PCOS group vs the control group (Fig. 1).

We further divided the PCOS group into four phenotypes according to the Rotterdam criteria: phenotype A (clinical and/or biochemical (HA) + oligo/amenorrhea (OA) + polycystic ovary morphology (PCOM); 18 women), phenotype B (HA + OA; 16 women), phenotype C (HA + PCOM; 13 women) and phenotype D (OA + PCOM; 10 women). We observed higher serum fetuin-B concentration in phenotypes C in comparison to the control group (*P* = 0.0006). We found higher plasma concentrations of TG (*P* = 0.02), as well as higher serum levels of LH (*P* = 0.0008), total testosterone (*P* = 0.0001), FAI (*P* < 0.01), glucose at 60′ (*P* < 0.01) and 120′ (*P* = 0.01) of OGTT, insulin at 120′ of OGTT (*P* = 0.001), GGT (*P* = 0.004), FLI (*P* = 0.007), LAP (*P* = 0.02) and VAI (*P* = 0.01) in phenotype A in comparison to the control group. In phenotype B, we found higher FAI (*P* = 0.002) and serum concentration of GGT (*P* = 0.007) in comparison to the control group. We also noticed higher serum GGT (*P* = 0.07), total testosterone concentration (*P* = 0.0001) and FAI (*P* = 0.002) in phenotype C in comparison to the control group (Table 2).

**Table 2:** Clinical and biochemical characteristics of the PCOS phenotypes.

	Control group (*n* = 51)	Phenotype A (*n* = 18)	Phenotype B (*n* = 16)	Phenotype C (*n* = 13)	Phenotype D (*n* = 10)
Age (years)	26.3 ± 4.3	25.5 ± 4.6	26.5 ± 5.2	24.4 ± 3.9	26.5 ± 5.4
BMI (kg/m^2^)	22.8 ± 3.1	22.8 ± 4.9	24.4 ± 3.3	23.8 ± 3.6	23.2 ± 3.1
Waist circumference (cm)	79.5 ± 8.3	84.1 ± 12.3	84.2 ± 9.6	80.4 ± 9.4	80.9 ± 8.5
Hip circumference (cm)	98.7 ± 7.1	99.3 ± 9.3	102.1 ± 4.0	98.7 ± 9.9	98.6 ± 7.6
Fat free mass (kg)	44.8 ± 3.7	44.5 ± 4.4	47.0 ± 8.6	44.8 ± 3.4	43.6 ± 3.9
Fat mass (%)	28.9 ± 6.3	32.9 ± 4.4	32.5 ± 9.4	29.9 ± 7.9	32.2 ± 7.5
Follicle-stimulating hormone (IU/L)	5.4 ± 1.7	6.3 ± 3.6	4.9 ± 1.7	4.4 ± 1.6	4.8 ± 1.3
Luteinizing hormone (IU/L)	4.1 ± 1.2	5.1 ± 1.5^a^	5.2 ± 2.2	5.8 ± 0.8	5.5 ± 1.3
Total testosterone (ng/mL)	0.56 ± 0.1	0.8 ± 0.2^a^	0.6 ± 0.2	0.7 ± 0.1^a^	0.5 ± 0.1
SHBG (nmol/L)	66.0 ± 30.2	42.2 ± 28.3^a^	43.2 ± 33.5^a^	56.2 ± 0.3^a^	85.9 ± 66.6
FAI	3.4 ± 2.5	8.7 ± 5.0^a^	6.3 ± 3.5^a^	7.0 ± 6.3^a^	2.7 ± 1.8
PRL (ng/mL)	15.5 ± 14.0	14.2 ± 11.9	12.0 ± 6.3	22.2 ± 19.1	14.9 ± 10.0
Glucose 0′ OGTT (mg/dL)	94.1 ± 9.5	93.5 ± 5.7	92.8 ± 7.4	90.2 ± 8.9	91.6 ± 6.3
Glucose 60′ OGTT (mg/dL)	97.7 ± 20.5	136 ± 39.1^a^	116 ± 37.9	99.4 ± 24.2	108 ± 35.8
Glucose 120′ OGTT (mg/dL)	88.8 ± 16.6	102 ± 26.2^a^	94.4 ± 19.5	93.7 ± 20.1	92.1 ± 22.6
Insulin 0′ OGTT (µIU/mL)	10.0 ± 4.8	10.2 ± 3.9	9.9 ± 4.1	10.2 ± 5.0	8.6 ± 3.2
Insulin 60′ OGTT (µIU/mL)	55.9 ± 45.8	84.6 ± 50.9	69.2 ± 42.3	64.8 ± 57.6	65.2 ± 23.5
Insulin 120′ OGTT (µIU/mL)	30. ± 18.3	74.4 ± 90.5^a^	29.6 ± 13.5	41.6 ± 34.5	37.1 ± 20.1
HOMA-IR	2.2 ± 1.1	2.4 ± 0.9	2.3 ± 1.0	2.3 ± 1.3	1.9 ± 0.8
HOMA-B	125 ± 70	123 ± 43	123 ± 56	140 ± 66	113 ± 56
Total cholesterol (mg/dL)	175 ± 28	174 ± 23	162 ± 18	163 ± 24	175 ± 31
HDL cholesterol (mg/dL)	65.7 ± 20.7	61.9 ± 21.2	59.4 ± 13.4	69 ± 10.2	71.2 ± 17.1
LDL cholesterol (mg/dL)	91.5 ± 24.3	95.9 ± 18.4	89.2 ± 23.3	82.2 ± 19.1	91.5 ± 26.1
TG (mg/dL)	62.0 ± 28.0	83.5 ± 62.4^a^	69.6 ± 39.7	60.1 ± 14.2	63.4 ± 21.0
Fetuin-B (µg/mL)	79.2 ± 16.7	88.1 ± 26.4	79.6 ± 14.8	98.9 ± 25.4^a^	82.5 ± 33.4
VAI	0.78 ± 0.48	1.5 ± 1.7^a^	1.1 ± 0.9	0.7 ± 0.2	0.7 ± 0.3
LAP	15.7 ± 10.7	28.7 ± 37.1^a^	22.8 ± 21.7	15.3 ± 7.4	17.3 ± 9.6
FLI	8.0 ± 10.2	20.0 ± 25.2^a^	16.4 ± 23.9	11.3 ± 16.4	9.9 ± 10.1
GGT	9.4 ± 2.7	12.4 ± 5.5^a^	14.0 ± 10.5^a^	13.4 ± 8.9^a^	12.9 ± 8.8^a^

Data are presented as mean ± s.d. The level of significance was accepted at ^a^*P* < 0.05 PCOS vs control group.

BMI, body mass index; FAI, free androgen index; FLI, fatty liver index; GGT, gamma-glutamyl transferase; HOMA-B; homeostatic model assessment β cell function; HOMA-IR, homeostasis model assessment of insulin resistance; LAP, lipid accumulation product; OGTT, oral glucose tolerance test; PCOS, polycystic ovary syndrome; SHBG, serum sex hormone–binding globulin; TG, triglycerides; VAI, visceral adiposity index.

We observed positive relationships between serum fetuin-B concentration and BMI (*r* = 0.32, *P* = 0.01), waist circumference (*r* = 0.30, *P* = 0.02) and percentage of body fat (*r* = 0.27, *P* = 0.04) in the PCOS group. We found a positive relationship between serum fetuin-B concentration and baseline serum insulin concentration (*r* = 0.36, *P* = 0.007), HOMA-IR (*r* = 0.31, *P* = 0.01) and HOMA-β (*r* = 0.43, *P* = 0.01), only in PCOS women. We also observed a relationship between serum fetuin-B concentration and plasma TG level (*r* = 0.30, *P* = 0.02) and FAI (*r* = 0.26, *P* = 0.04) in the PCOS group (Table 3). We found a positive association between serum fetuin-B concentration and FLI (*r* = 0.29, *P* = 0.02), VAI (*r* = 0.29, *P* = 0.02) and LAP (*r* = 0.32, *P* = 0.01), only in the PCOS group. Multiple regression analysis revealed that HOMA-B (β = 0.39, *P* = 0.002) and LAP (β = 0.27, *P* = 0.02) were independently connected with serum fetuin-B levels in women with PCOS (Table 4).

**Table 3:** Correlation between serum concentration of fetuin-B with anthropometric and biochemical parameters in PCOS and control group.

	PCOS (*n* = 57)	Control (*n* = 51)
BMI	*r* = 0.32, *P* = 0.01	*r* = 0.33, *P* = 0.01
Waist circumference	*r* = 0.30, *P* = 0.02	*r* = 0.19, *P* = 0.16
Fat mass (%)	*r* = 0.27, *P* = 0.04	*r* = 0.12, *P* = 0.39
Fat free mass (kg)	*r* = 0.14, *P* = 0.30	*r* = 0.30, *P* = 0.02
Baseline insulin	*r* = 0.36, *P* = 0.007	*r* = 0.09, *P* = 0.6
HOMA-IR	*r* = 0.31, *P* = 0.01	*r* = 0.07, *P* = 0.6
HOMA-B	*r* = 0.43, *P* = 0.01	*r* = 0.66, *P* = 0.06
TG	*r* = 0.30, *P* = 0.02	*r* = 0.22, *P* = 0.12
FAI	*r* = 0.26, *P* = 0.04	*r* = 0.33, *P* = 0.01
FLI	*r* = 0.29, *P* = 0.02	*r* = 0.24, *P* = 0.08
VAI	*r* = 0.29, *P* = 0.02	*r* = 0.21, *P* = 0.13
LAP	*r* = 0.32, *P* = 0.01	*r* = 0.25, *P* = 0.69

Data are derived from Spearman’s rank correlation. The level of significance was accepted at *P* < 0.05.

FAI, free androgen index; FLI, fatty liver index; HOMA-B, homeostasis model assessment β cell function; HOMA-IR, homeostasis model assessment of insulin resistance; LAP, lipid accumulation product; PCOS, polycystic ovary syndrome; TG, triglycerides; VAI, visceral adiposity index.

**Table 4:** Univariate and multivariable regression analysis with fetuin-B as a dependent variable in PCOS group.

Variables	Univariate		Multivariate	
	β (95% CI)	*P* value	β (95% CI)	*P* value
HOMA-β	0.44 (0.19–0.68)	0.0007	0.39 (0.15–0.63)	0.002
LAP	0.32 (0.07–0.58)	0.01	0.27 (0.03–0.51)	0.02

HOMA-β, homeostasis model assessment β cell function; LAP, lipid accumulation product.

**Table 5:** Correlation between indices of fatty liver steatosis with anthropometric, biochemical and hormonal parameters in PCOS group.

	PCOS (*n* = 57)		
	FLI	LAP	VAI
Percentage of body fat	*r* = 0.8, *P* < 0.01	*r* = 0.51, *P* < 0.01	*r* = 0.3, *P* = 0.03
HOMA-IR	*r* = 0.42, *P* = 0.01	*r* = 0.41, *P* = 0.001	*r* = 0.38, *P* = 0.004
HDL cholesterol	*r* = −0.33, *P* = 0.01	*r* = −0.33, *P* = 0.01	NE
FAI	*r* = 0.51, *P* < 0.01	*r* = 0.39, *P* = 0.002	*r* = 0.31, *P* = 0.01

Data are derived from Spearman’s rank correlation. The level of significance was accepted at *P* < 0.05.

FAI, free androgen index; FLI, fatty liver index; HOMA-IR, homeostasis model assessment of insulin resistance; LAP, lipid accumulation product; NE, not estimated; PCOS, polycystic ovary syndrome; VAI, visceral adiposity index.

We observed a positive relationship between HOMA-IR and FLI (*r* = 0.42, *P* = 0.01), VAI (*r* = 0.38, *P* = 0.004) and LAP (*r* = 0.41, *P* = 0.001) and a negative correlation between FLI and plasma HDL-C concentration (*r* = −0.33, *P* = 0.01), as well as between LAP and level of HDL cholesterol (*r* = −0.33, *P* = 0.01) in PCOS women. We found a relationship between percentage of body fat and FLI (*r* = 0.8, *P* < 0.01), VAI (*r* = 0.3, *P* = 0.03) and LAP (*r* = 0.51, *P* < 0.01) and between FAI and FLI, VAI and LAP (*r* = 0.51, *P* < 0.01; *r* = 0.31, *P* = 0.01; *r* = 0.39, *P* = 0.002, respectively) in PCOS women (Table 5).

In the control group, there were positive correlations between serum fetuin-B concentration and BMI (*r* = 0.33, *P* = 0.01), fat free mass (*r* = 0.30, *P* = 0.02) and with FAI (*r* = 0.33, *P* = 0.01) (Table 3). Additionally, we found a positive relationship between FLI and percentage of body fat (*r* = 0.6, *P* < 0.01) and HOMA-IR (*r* = 0.75, *P* = 0.001), as well as between LAP and HOMA-IR (*r* = 0.4, *P* = 0.03) in this group. We also noticed a relationship between FAI and FLI (*r* = 0.63, *P* < 0.01) and LAP (*r* = 0.37, *P* = 0.007) in this group.

We did not find any correlation between serum fetuin-B and LH, FSH, as well as total testosterone concentration in both groups (all *P* > 0.05).

## Discussion

In the current study, we showed higher serum fetuin-B concentration and FLI in women with PCOS in comparison to the control group matched for BMI and age. We also presented a relationship between serum levels of fetuin-B and HOMA-IR, HOMA-β, as well as different indices of liver steatosis, that is, FLI, VAI and LAP in PCOS women. Accordingly, we observed that HOMA-β and LAP were independent predictors of circulating fetuin-B in PCOS women. Interestingly, when we divided PCOS group into phenotypes according to the Rotterdam criteria, we observed higher serum fetuin-B concentrations in phenotype C in comparison to the control group. Accordingly, indices of liver steatosis were higher in phenotypes A in comparison to the control group. However, based on a small number of subjects, it would be premature to draw any conclusions.

Several case–control and cross-sectional studies have demonstrated that the frequency of NAFLD is increased in women with PCOS, independent of overweight/obesity and other coexisting metabolic syndrome features (5, 21, 22). Additionally, it has been presented that PCOS women with NAFLD are characterized by higher degree of insulin resistance and higher prevalence of metabolic syndrome (21). As it was mentioned in the Introduction section, it has been shown that serum fetuin-B is increased in NAFLD and may be connected with insulin resistance (11). However, the factors linking NAFLD with lower insulin sensitivity are not fully understood, especially in PCOS women (4, 21). In our study, we observed higher serum fetuin-B concentration and FLI as well as a relationship between serum fetuin-B concentration and different indices of liver steatosis and insulin resistance in PCOS women. Moreover, in a multivariate analysis we observed that LAP was an independent predictor of serum concentration of fetuin-B in this group. Therefore, based on our results, it might be suggested that fetuin-B could be one of the factors connected with NAFLD in PCOS women, probably by enhancing insulin resistance. The mechanism how fetuin-B might influence insulin action is not fully understood. As it was previously mentioned, the possible explanation came from the experimental study by Meex *et al*. demonstrating that fetuin-B impairs insulin action in myotubes and hepatocytes in mice (8). Therefore, we can hypothesize that increased serum synthesis of fetuin-B observed in NAFLD could be linked to decreased insulin sensitivity in muscle and liver. However, the role of fetuin-B in PCOS women is still unclear and requires further investigations. Based on the obtained results, we can suggest that serum fetuin-B concentration could be proposed as a biomarker of NAFLD or could reflect the risk of development of liver steatosis in early stage in PCOS women. On the contrary, Ebert *et al*. did not observe a relationship between markers of liver fibrosis in NAFLD and fetuin-B. Nevertheless, they examined patients with advanced stages of NAFLD, and the researchers speculated that fetuin-B could be involved in the natural course of NAFLD progression. Therefore, reduced hepatokine synthesis in advanced NAFLD could be observed (23).

Importantly, we observed that HOMA-β and LAP were independent predictors of circulating fetuin-B in PCOS women. To the best of our knowledge, only one study evaluated the relationship between serum concentration of fetuin-B and HOMA-β. The authors found a negative correlation between the mentioned factors (24). Accordingly, they also observed that serum fetuin-B is negatively correlated with first-phase glucose-stimulated insulin secretion estimated by intravenous glucose tolerance test. On the contrary, we found a positive relationship between serum fetuin-B concentration and HOMA-β. The obtained difference could be associated with a different studied group. In the mentioned study, the participants had diabetes or prediabetes, in which the observed impairment of insulin secretion, especially first-phase secretion, is more severe than in young PCOS women. Our findings are in agreement with other data focused on the relationships between serum fetuin-B concentration and different states of insulin resistance (8, 12, 13). The mentioned researchers found that serum fetuin-B is connected with fasting insulin, as well as HOMA-IR (8). In another study, Li *et al*. reported that subjects with T2D or NAFLD showed significantly increased serum fetuin-B levels compared to the control group (12). The authors concluded that fetuin-B links NAFLD to T2D via inducing insulin resistance (12). In another study, it has been shown that serum fetuin-B concentrations were significantly higher in patients with T2DM compared to patients with normal glucose tolerance test and prediabetes and positively correlated with plasma glucose in the fasting state and 2 h after meal, fasting insulin, HOMA-IR, first-phase glucose-stimulated insulin and TG (24). On the contrary, recently published data did not demonstrate a relationship between HOMA-IR and hepatic mRNA expression of fetuin-B. However, the authors examined a heterogeneous group, that is men and women with median age of 65 years, some of whom had diabetes (25).

In a study performed by Macut *et al*., a relationship between NAFLD liver fat score and waist circumference, BMI, baseline glucose, HOMA-IR, TG and LAP was shown, whereas in a multivariate logistic regression analysis, HOMA-IR and LAP were independently associated with NAFLD (21). In our study, we also observed a relationship between indices of liver steatosis and percent of body fat, HOMA-IR and HDL cholesterol in PCOS women. Similarly, in the previous study of the aforementioned authors, a correlation between LAP and the presence of insulin resistance, metabolic syndrome and impaired glucose tolerance in PCOS women has been shown (5, 26). All of these findings might have a therapeutic implication. It has been shown that administration of metformin could improve IR as well as NAFLD in PCOS women (27). However, there are no data evaluating the impact of metformin, as well as diet or physical exercise, on serum level of fetuin-B in PCOS women.

Moreover, we also observed a relationship between FAI and indices of liver steatosis (FLI, VAI and LAP) in PCOS women. The previously published studies showed conflicting results. Some authors reported lack of association between NAFLD liver fat score and androgens in PCOS women (5), whereas others showed an independent correlation (28, 29). Interestingly, elevated serum levels of ALT and GGT in overweight/obese PCOS women compared with overweight/obese controls have been demonstrated. However, the authors did not find any difference in serum liver enzyme between lean PCOS and lean controls. Accordingly, they observed a positive relationship between serum levels of ALT and FAI in overweight/obese PCOS patients (30).

A few limitations in the present study should be recognized. First, the analysis of serum fetuin-B concentrations has been performed in a cross-sectional design, and, therefore, causality cannot be established. Second, NAFLD was determined by FLI and other indirect indices, and hepatic ultrasonography scanning or MRS was not performed in the studied group. However, it has been shown that ultrasound might underestimate the prevalence of liver steatosis (31) and FLI is a good marker of liver steatosis in PCOS women (32). Additionally, FLI was cross-validated in large studies with sensitivity and specificity of 80.3 and 87.3%, respectively, in comparison to ultrasound (33). Moreover, in our study, we used measures of liver fat derived from well-validated algorithms (5, 20, 32, 33, 34). Another possible limitation of the present study could be the measurement of total testosterone by RIA and not with the liquid chromatography-tandem mass spectrometry (LC-MS) method. However, LC-MS is not widely used due to the costs of the measurements. Moreover, it should also be mentioned that the ‘gold standard’ for the measurement of insulin sensitivity is hyperinsulinemic–normoglycemic clamp technique, whereas we used HOMA-IR which have been found to be inaccurate for the assessment of insulin sensitivity in women with PCOS (35, 36).

## Conclusions

We concluded that serum fetuin-B levels are higher in women with PCOS and are independently connected with HOMA-β and hepatic steatosis. Therefore, serum fetuin-B concentration could be proposed as a biomarker of NAFLD and might reflect the risk of development of liver steatosis at an early stage in PCOS women.

## Declaration of interest

The authors declare that there is no conflict of interest that could be perceived as prejudicing the impartiality of the research reported.

## Funding

This work was supported by the Medical University of Białystok (grant number N/ST/ZB/17/007/1150).

## Author contribution statement

A A: conception and design of the study, acquisition of data, analysis and interpretation of data, writing the article; A M P, A Ł, A K, J H, M L: acquisition of data; I K: analysis and interpretation of data, revising the article, final approval of the version to be submitted.
